# Wide field of view and full Stokes polarization imaging using metasurfaces inspired by the stomatopod eye

**DOI:** 10.1515/nanoph-2022-0712

**Published:** 2023-02-24

**Authors:** Jianying Liu, Jinkui Chu, Ran Zhang, Rui Liu, Jiaxin Fu

**Affiliations:** Key Laboratory for Micro/Nano Technology and System of Liaoning Province, Dalian University of Technology, Dalian, China

**Keywords:** bionic compound eye, full Stokes polarization imaging, metasurfaces, wide field of view

## Abstract

Wide field of view and polarization imaging capabilities are crucial for implementation of advanced imaging devices. However, there are still great challenges in the integration of such optical systems. Here, we report a bionic compound eye metasurface that can realize full Stokes polarization imaging in a wide field of view. The bionic compound eye metasurface consists of a bifocal metalens array in which every three bifocal metalenses form a subeye. The phase of the bifocal metalens is composed of gradient phase and hyperbolic phase. Numerical simulations show that the bifocal metalens can not only improve the focusing efficiency in the oblique light but also correct the aberration caused by the oblique incident light. And the field of view of the bionic compound eye metasurface can reach 120° × 120°. We fabricated a bionic compound eye metasurface which consists of three subeyes. Experiments show that the bionic compound eye metasurface can perform near diffraction-limited polarization focusing and imaging in a large field of view. The design method is generic and can be used to design metasurfaces with different materials and wavelengths. It has great potential in the field of robot polarization vision and polarization detection.

## 1 Introduction

The compound eyes of stomatopods are a complex multichannel optical system. Compared with traditional optical systems, it has the advantages of small size, large field of view, high time sensitivity and having polarized vision [1–4]. Generally, the bionic compound eyes are designed as a planar microlens array or a curved microlens array [5–10]. The disadvantage of planar microlens arrays is the small field of view [11–13]. Although the curved microlens array has a large field of view, the incompatibility between the curved microlens array and the planar detector leads to defocusing problem [14, 15]. The method of gluing microlenses and photodetectors solves the alignment problem between the focal points of the curved microlens array and the detectors, but the size of the bionic compound eyes made by this method is larger than that of the natural compound eyes [16, 17]. In addition, the current research on the bionic compound eyes with large field of view neglects the polarized vision of the natural compound eyes.

Metasurfaces have the ability to control the phase and polarization of light in the subwavelength range and have great potential for the integration of optical devices [18–21]. For example, metasurfaces have been widely used to design metalens with polarization functions [22–25]. The research of polarization imaging based on dielectric metasurfaces mainly includes multispectral chirality imaging [26], Fourier matrix optics [27], and imaging polarimetry [28]. However, related studies on polarized imaging metasurfaces have the disadvantage of small field of view. The methods to eliminate the large field of view aberrations of dielectric metasurfaces mainly include: using a double-layer metasurface to correct the aberrations [29]; designing metalens with fisheye lens function [30]; relaxing the constraint on diffraction-limited resolution [31]; using metalens arrays [32]. The combination of gradient phase and hyperbolic phase has been demonstrated to be an effective method for increasing the field of view of metalenses for unpolarized light [32]. The disadvantage of these methods is that they are not compatible with the design of polarized imaging metasurfaces. In addition, related studies have shown that bifocal metalens array with polarization multiplexing can be used to expand the field of view [33, 34]. However, the metasurface still does not realize full Stokes polarization imaging in a large field of view.

Inspired by the compound eyes of stomatopods, the paper demonstrates a bionic compound eye metasurface (BCEM) with full Stokes polarization imaging in a large field of view. The BCEM consists of a bifocal metalens array in which every three bifocal metalenses form a sub eye. Each sub eye can decompose the incident light into two pairs of linearly polarized light and one pair of circularly polarized light, and these are used to reconstruct the Stokes parameters. The orientation of the visual axis is different for each sub eye, so the sub eye array can perform full Stokes polarization imaging of targets in different directions. The phase of the bifocal metalens is composed of gradient phase and hyperbolic phase. The function of gradient phase and hyperbolic phase are to correct the light vector in the oblique direction to the vertical direction and modulate the wavefront of the light, respectively. The time-domain finite-difference (FDTD) method was used to numerically simulate the effect of gradient phase on the transmittance and focusing efficiency, and the ability to correct off-axis aberration. We fabricated a BCEM consisting of three sub eyes, and each sub eye has a different gradient phase. After characterizing the modulation transfer function (MTF) and focusing efficiency, etc. of the bifocal metalens in the sub eye, we experimentally achieved full Stokes polarization imaging of the BCEM in a large field of view.

## 2 Design of BCEM

**Figure 1:**
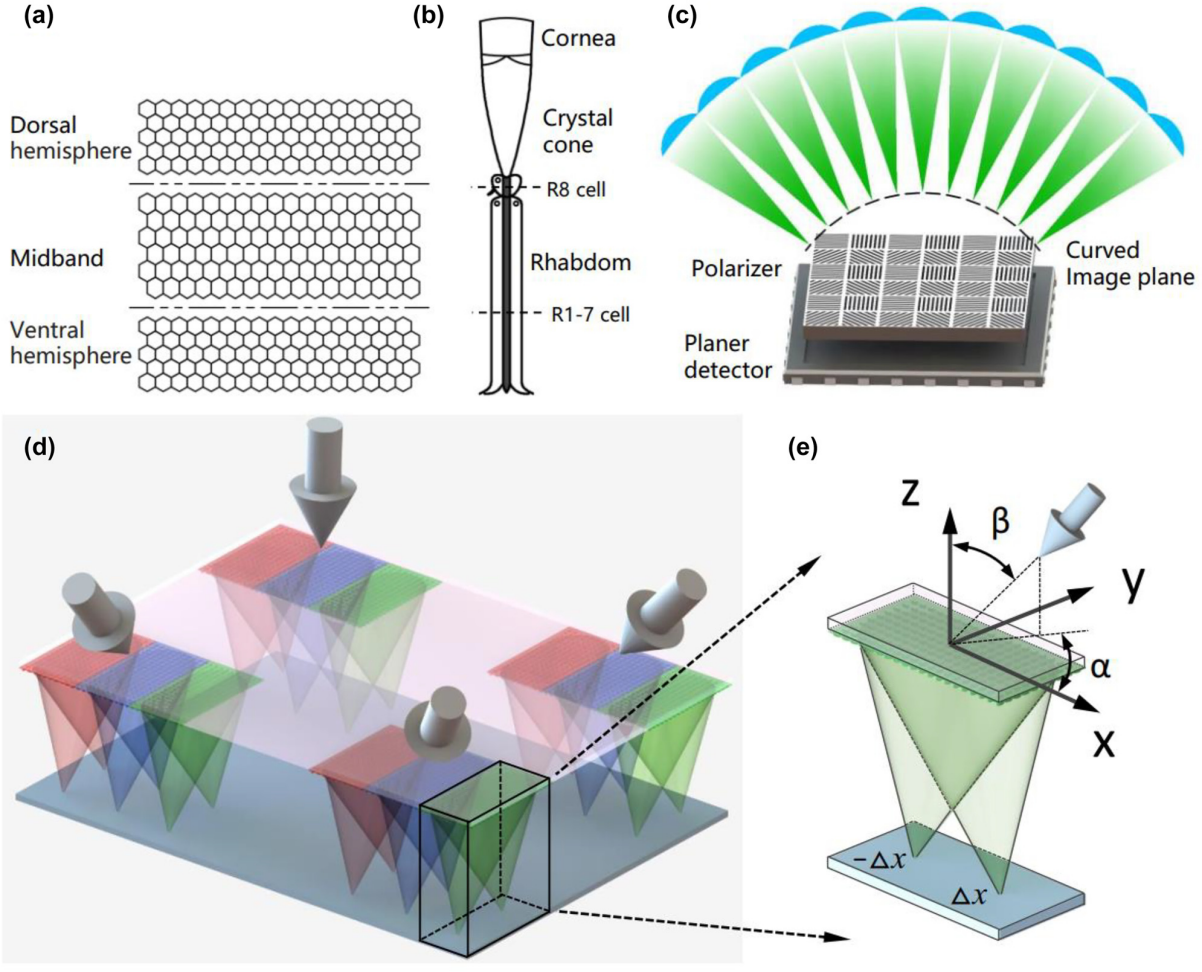
Schemes of BCEM. (a) Schematic diagram of mantis shrimp’s ommatidium distribution. (b) Anatomical schematic of ommatidium in the compound eye of mantis shrimp (adapted from [36]). (c) Schematic diagram of a bionic compound eye with polarized vision; there is a mismatch between the curved image surface and the polarizer and planar detector. (d) Schematic diagram of the BCEM. (e) Schematic diagram of the bifocal metalens in a sub eye, where*α* and *β* are the azimuth and elevation angles of the sub eye visual axis, respectively.

The compound eye of stomatopods (e.g., mantis shrimps) is a complex polarized optical system, which consists of six row of midband and dorsal and ventral hemispheres [35], and the distribution of ommatidium as shown in Figure 1(a). Each ommatidium consists of cornea, crystal cone and rhabdom, as shown in Figure 1(b), where the retinal cells (R8) and (R1-7) are sensitive to polarized light. Currently, a schematic diagram of the bionic compound eye with polarized vision is shown in Figure 1(c). The performance of the bionic compound eye can be severely affected since the curved imaging plane is mismatched with the polarizer and the planar detector [2]. To solve the problem, we use metasurfaces instead of traditional microlens arrays to design the bionic compound eyes. In this paper, we propose a BCEM with full Stokes polarization imaging function in a wide field of view, the schematic diagram is shown in Figure 1(d). The BCEM consists of a bifocal metalens array, where three bifocal metalens form a sub eye. The three bifocal metalens that make up the sub eye are denoted by the symbols X/Y, 45/135 and R/L, which decompose the incident light into X-LP and Y-LP (0° and 90° linearly polarized light), 45°-LP and 135°-LP (45° and 135° linearly polarized light), RCP and LCP (right-handed and left-handed circularly polarized light).

**Figure 2:**
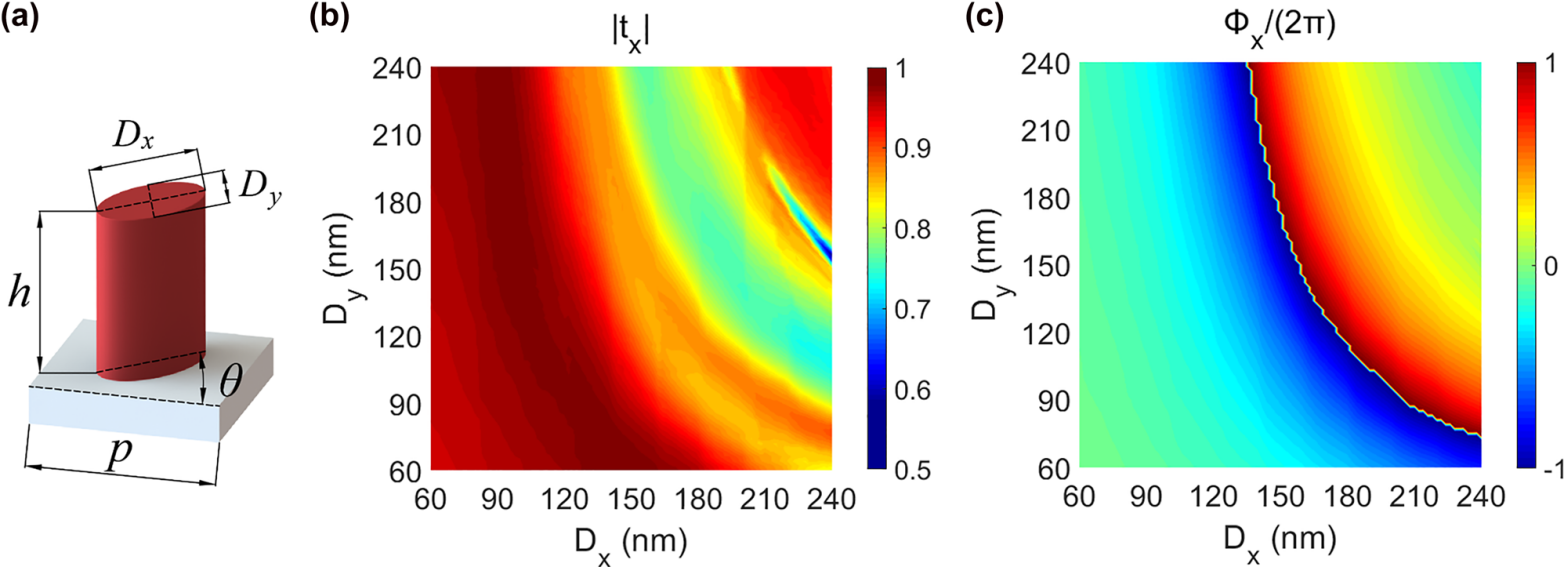
Design of the metasurface. (a) Schematic diagram of the elliptical silicon pillar, the substrate is silica. (b) and (c) Calculated transmittance and phase shift of a single period for X-LP normal incident light with a wavelength of 780 nm.

The azimuthal angle *α* and elevation angle *β* are used to describe the orientation of the sub eye visual axis, as shown in Figure 1(e). The coordinates of the sub eye in the bionic compound eye metasurface are also represented by the azimuth and elevation angles, namely (*α*, *β*). The metasurface consists of a series of elliptical silicon pillars with height *h* = 400 nm and period *p* = 300 nm, as shown in Figure 2(a). We use the FDTD method to numerically simulate the effects of the major axis *D*_*x*_ and minor axis *D*_*y*_ on the phase shift and transmittance within a single period, as shown in Figure 2(b) and (c).

In order to realize the function of simultaneous polarization imaging of targets in different directions with BCEM, the phase of the bifocal metalens uses a combination of gradient phase and hyperbolic phase, where the gradient phase is used to correct the light vector in the oblique direction to the vertical direction. If the azimuth and elevation angles of the sub eye visual axis are *α* and *β*, as shown in Figure 1(b). Then the gradient phase of the sub eye can be described as:

(1) $$\varphi_{\left(\alpha ,\beta \right)}=\frac{2\pi }{\lambda }\sin\left(\beta \right)\sqrt{{\left(x\cos\left(\alpha \right)\right)}^{2}+{\left(y\sin\left(\alpha \right)\right)}^{2}}$$

where *α* and *β* are the azimuth and elevation angles, *λ* is the wavelength of light, $\sin\left(\beta \right)\sqrt{{\left(x\cos\left(\alpha \right)\right)}^{2}+{\left(y\sin\left(\alpha \right)\right)}^{2}}$ is the optical path difference between the oblique direction and the vertical direction of the light vector.

The hyperbolic phase of the bifocal metalens is used to control the wavefront of light and focus it [37]. If the distance of the two focal points from the center of the bifocal metalens is ±Δ*x*. Then the hyperbolic phase of the bifocal metalens satisfies:

(2) $$\varphi_{\pm \Delta x}=-\frac{2\pi }{\lambda }\left(\sqrt{(x\pm \Delta {x)}^{2}+y^{2}+f^{2}}-f\right)$$

where *f* is the focal length of the bifocal metalens.

The phase of the bifocal metalens is a combination of the gradient phase and hyperbolic phase, as shown in Equation (3).

(3) $$\begin{array}{ll} \varphi_{\left(\alpha ,\beta \right)\pm \Delta x} & =-\frac{2\pi }{\lambda }\left(\sqrt{(x\pm \Delta {x)}^{2}+y^{2}+f^{2}}-f\right.\\ & \;\left.-\sin\left(\beta \right)\sqrt{{\left(x\cos\left(\alpha \right)\right)}^{2}+{\left(y\sin\left(\alpha \right)\right)}^{2}}\right) \end{array}$$

It can be seen from Figure 2(b) and (c) that the phase shift is continuous from 0 to 2π. The independent control of the orthogonal line polarization light by the bifocal metalens can be achieved by selecting the suitable size of the elliptical pillars [38]. Therefore, the parameters of the X/Y and 45/135 bifocal metalenses can be calculated using Equation (3). The parameters of the R/L bifocal metalens are calculated using the improved PB phase method [38], as shown in Equations (4) and (5).

(4) $$E_{r}^{\text{out}}={\text{e}}^{\text{i}\Delta \varphi_{r}}\left[\frac{{\text{e}}^{\text{i}\varphi_{x}}+{\text{e}}^{\text{i}\varphi_{y}}}{2}\left[\begin{array}{c} 1\\ i \end{array}\right]+\frac{{\text{e}}^{\text{i}\varphi_{x}}-{\text{e}}^{\text{i}\varphi_{y}}}{2}\left[\begin{array}{c} 1\\ -i \end{array}\right]e^{i2\theta }\right]$$

(5) $$E_{l}^{\text{out}}={\text{e}}^{\text{i}\Delta \varphi_{l}}\left[\frac{{\text{e}}^{\text{i}\varphi_{x}}+{\text{e}}^{\text{i}\varphi_{y}}}{2}\left[\begin{array}{c} 1\\ -i \end{array}\right]+\frac{{\text{e}}^{\text{i}\varphi_{x}}-{\text{e}}^{\text{i}\varphi_{y}}}{2}\left[\begin{array}{c} 1\\ i \end{array}\right]e^{-i2\theta }\right]$$

where, $E_{r}^{\text{out}}$ and $E_{l}^{\text{out}}$ are the right-handed and left-handed circularly polarization components of the emitted light, respectively. ${\text{e}}^{\text{i}\Delta \varphi_{r}}$ and ${\text{e}}^{\text{i}\Delta \varphi_{l}}$ are the additional phases of the orthogonal circular polarization components of the incident light. ${\text{e}}^{\text{i}\varphi_{x}}$ and ${\text{e}}^{\text{i}\varphi_{y}}$ are the phase shift of the elliptical pillars in the *X* and *Y* directions, respectively. *θ* is the rotation angle of the elliptical pillars. The elliptical pillar acting on circularly polarized light is regarded as a half-wave plate, so Equations (4) and (5) also indicate that the chirality of the light is reversed by the bifocal metalens.

## 3 Numerical simulation and performance of bifocal metalens

**Figure 3:**
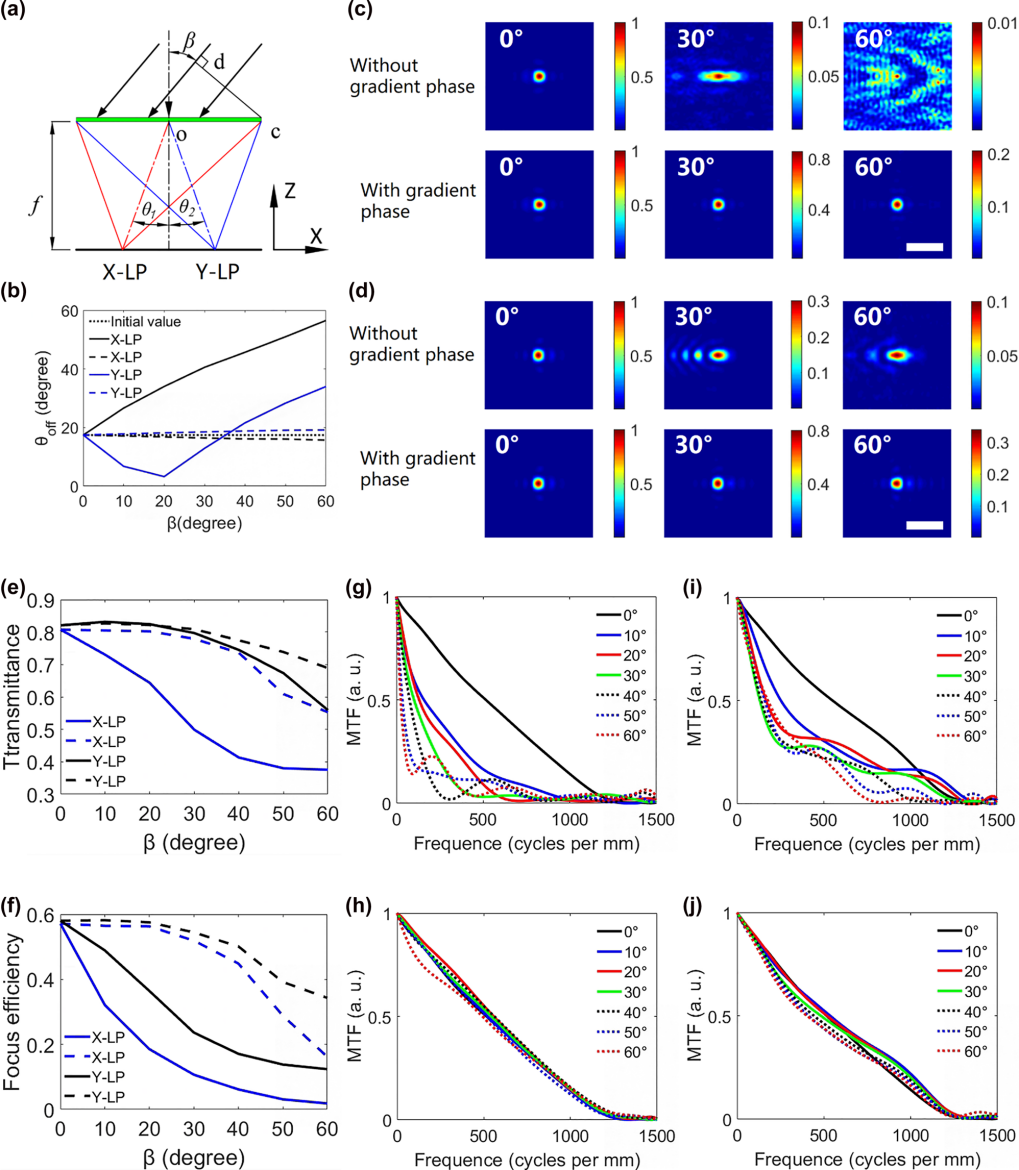
Theoretical analysis and numerical simulation of the metalens. (a) Schematic diagram of the optical path of the bifocal metalens in the XOZ plane, where *θ*_1_ and *θ*_2_ are the off-axis angles of the X-LP and Y-LP components, respectively. The line cd represents the optical path difference between the oblique light vector and the vertical light vector on the bifocal metalens. (b) When *α* = 0, the off-axis angles of the X-LP and Y-LP components in (a), where the solid and dashed lines represent the traditional bifocal metalens and the bifocal metalens with gradient phase, respectively, and the dotted line represents the initial value. (c) and (d) When *α* = 0, the focused spots of X-LP and Y-LP components of the two bifocal metalenses at different elevation angles, respectively. Scale bar: 3 μm. (e) and (f) When *α* = 0, the transmittance and focusing efficiency of the two bifocal metalenses, the solid and dashed lines represent the traditional bifocal metalens and the bifocal metalens with gradient phase, respectively. (g) and (h) MTFs of the X-LP components at different elevation angles for the traditional bifocal metalens and the bifocal metalens with gradient phase, respectively. (i) and (j) MTFs of the Y-LP components at different elevation angles for the traditional bifocal metalens and the bifocal metalens with gradient phase, respectively.

Taking X/Y bifocal metalens as an example, we use the FDTD method to simulate the correction of off-axis aberration by the gradient phase. The size of the bifocal metalens is 15 × 30 μm, and the focal length is 24.2 μm. In the paper, the angle corresponding to the distance from the spot position to the center of the bifocal metalens is defined as the off-axis angle, as shown in Figure 3(a). In the simulation, the azimuth angle is 0°, the elevation angle is changed from 0° to 60°, and the incident light is linearly polarized light (X-LP and Y-LP). The *θ*_1_ and *θ*_2_ in Figure 3(a) are the off-axis angles of the X-LP and Y-LP components, respectively. For a traditional bifocal metalens, the two focal points in Figure 3(a) shift to the left as the elevation angle *β* increases. The curves of off-axis angles *θ*_1_ and *θ*_2_ with the elevation angle are shown as solid lines in Figure 3(b). For the bifocal metalens with gradient phase, the azimuth and elevation angles of metalens are equal to the angle of the incident light. Thus the gradient phase compensates the optical path difference caused by the oblique light, and the off-axis angles of the two focal points remain near their initial values, as shown by the dashed lines in Figure 3(b). Figure 3(c) and (d) show the focused spots of the X-LP and Y-LP components of the two bifocal metalenses at different elevation angles, respectively. It can be seen from Figure 3(b)–(d) that the aberration of traditional bifocals increases with the off-axis angle increases, which will reduce the imaging resolution. However, the off-axis angle and aberration of the bifocal metalens with gradient phase are almost unchanged. Thus, it can be concluded that the gradient phase can keep the off-axis angle constant at oblique incident light and reduce the aberration of the bifocal metalens. In addition, the constant off-axis angle avoids light crosstalk from the adjacent bifocal metalenses with different visual axes. This characteristic of the gradient phase is used to design the BCEM with a large field of view.

We also simulated the transmittance and focusing efficiency of the two bifocal metalenses, as shown in Figure 3(e) and (f). It can be seen from these figures that the bifocal metalens with gradient phase has higher transmittance and focusing efficiency when the elevation angle increases. The X-directional MTF of the bifocal metalens is the Fourier transform of the X-directional line spread function (LSF). Figure 3(g), (h) and (i), (j) show the MTFs of the X-LP and Y-LP components in the X-direction for the two bifocal metalenses, respectively. It can be seen from these figures that the MTFs of the bifocal metalens with gradient phasing is higher than that of the traditional bifocal metalens when the elevation angle increases. Therefore, the same conclusion as in the previous paragraph can be deduced that the gradient phase can reduce the aberration caused by oblique incident light.

## 4 Characterization and polarization imaging performance of BCEM

**Figure 4:**
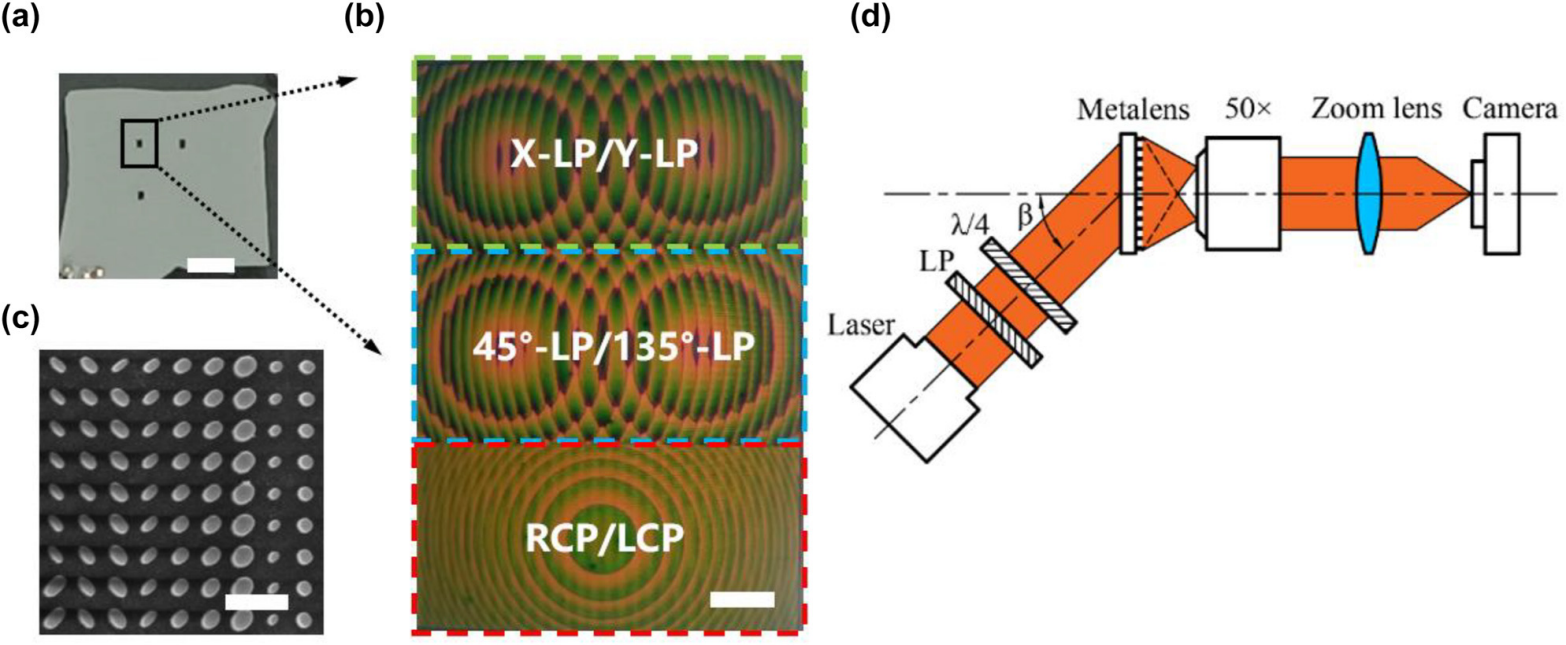
Manufactured BCEM sample and its test optical path. (a) Photograph of the BCEM sample. Scale bar: 1 mm. (b) Microscopic image of the sub eye, which consists of three bifocal metalenses. Three bifocal metalenses decompose the light into X-LP and Y-LP, 45°-LP and 135°-LP, RCP, and LCP, respectively. Scale bar: 20 μm. (c) SEM image of the bionic compound eye metasurface. Scale bar: 600 nm. (d) Schematic diagram of the optical path for testing the focusing performance of the BCEM. Where LP and λ/4 denote line polarizer and quarter-wave plate.

We fabricated a BCEM with three sub eyes whose coordinates are (0,0), (0,30), and (90,30), respectively. The size of the sub eye is 120 × 180 μm, and the focal length is 340 μm. To reduce the effect of stray light on the experiment, we fabricated 200 nm chromium (Cr) around the sub eyes as a light shielding film, and its fabrication process in Supplementary Materials Section 2. Photograph of the BCEM sample is shown in Figure 4(a), and its microscope and scanning electron microscope (SEM) images are shown in Figure 4(b) and 4(c), respectively.

The schematic diagram of the polarization focusing experiment of the BCEM is shown in Figure 4(d). A collimated laser (Fulei, China, FU780AD100-PXG2698-20) with a wavelength of 780 nm is used as the light source, and the laser is passed through a linear polarizer (Codixx, Germany, MPF10-600-10,000) and a quarter-wave plate (THORLABS, AQWP05M-580) to form polarized light with a specific polarization state. Then, a 50× objective lens (Mitutoyo, M Plan Apo 50X/0.55), a zoom lens (navitar-1 6010) and a camera (Sony, Basler acA4024-29um) were used to magnify the image formed by the BCEM. The angle between the direction of the light source and the metasurface is *β*, which is equal to the elevation angle of the sub eye in the experiment.

It can be known from the previous section that the sub eye located at (0,0) acts on the normal incident light with a value of zero for the gradient phase. Therefore, the test results of this sub eye in oblique light can be used for the comparison data of the other two sub eyes. First we tested the polarization focusing performance of the X/Y bifocal metalenses in the sub eyes and calculated the corresponding MTFs, where the light source was set to linearly polarized light (X-LP and Y-LP).

**Figure 5:**
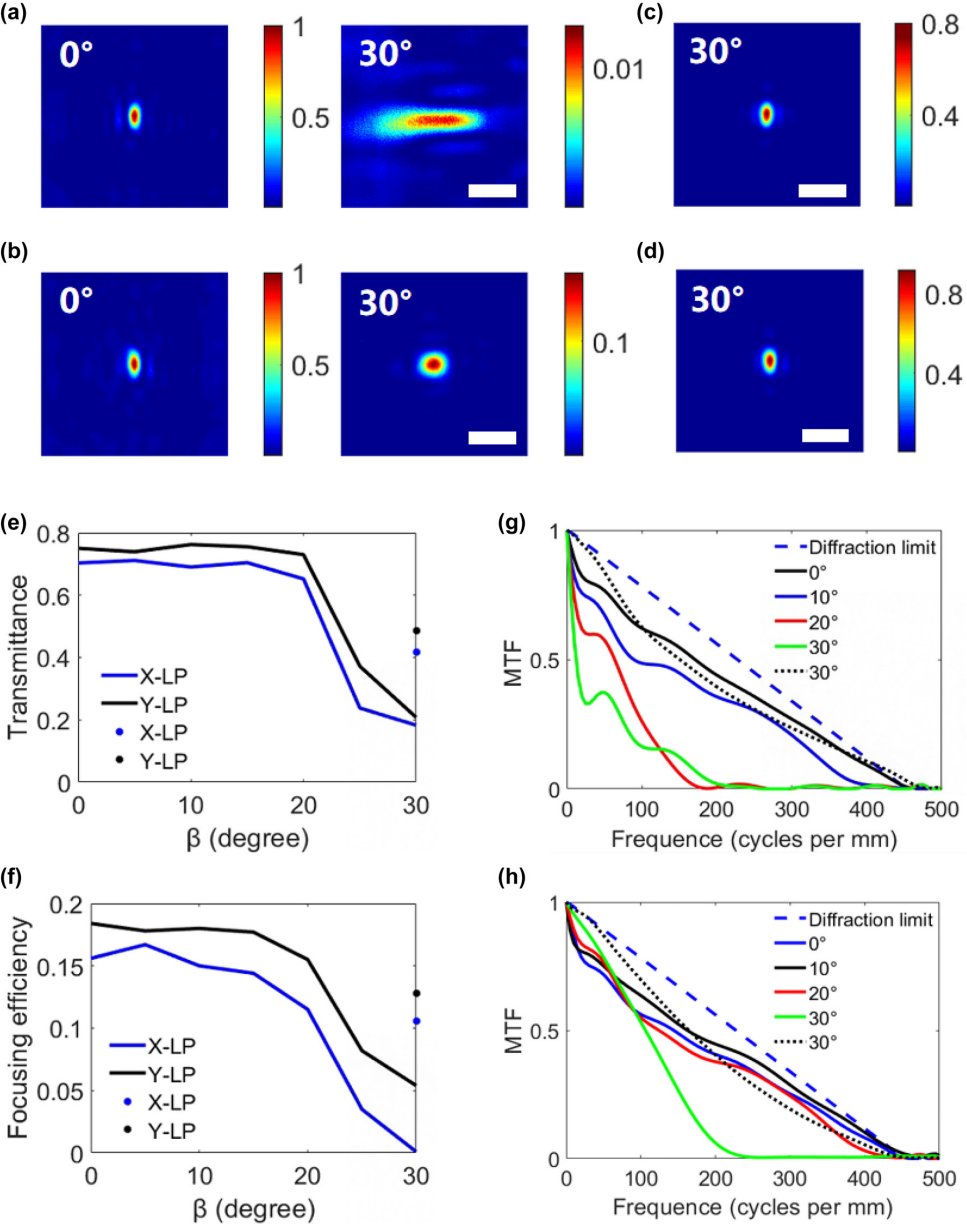
Experiment of polarization focusing by the metalens. (a) and (b) The focused spot of the X-LP and Y-LP components of the bifocal metalens located at (0,0), respectively. (c) and (d) The focused spot of the X-LP and Y-LP components of the bifocal metalens located at (0,30), respectively. The angle in (a)–(d) is the value of *β* in the experiment. Scale bar 10 μm. (e) and (f) Transmittance and focusing efficiency of the X/Y bifocal metalenses. The solid line and dots represent the X/Y bifocal metalens located at (0,0) and (0,30), respectively. (g) and (h) MTFs of X-LP and Y-LP components of the X/Y bifocal metalenses, where the solid and dashed lines represent the X/Y bifocal metalens located at (0,0) and (0,30), respectively, and the dashed line represents the diffraction limit.

The measurements of the focused spot for the X/Y bifocal metalenses located at (0,0) and (0,30) are shown in Figure 5(a)–(d). Figure 5(a) and (b) show the focused spots of the X-LP and Y-LP components of the bifocal metalens located at (0,0), respectively. As can be seen from Figure 3(b), the off-axis angle of the X-LP component is larger than that of the Y-LP component when *β* = 30°. Similar to the simulated results, the aberration of the X-LP component is larger than that of the Y-LP component for the bifocal metalens located at (0,0) when *β* = 30°. Figure 5(c) and (d) show the focused spots of the X-LP and Y-LP components of the bifocal metalens located at (0,30), respectively. Compared with Figure 5(a) and (b), Figure 5(c) and (d) show the correction of off-axis aberration caused by oblique incident light corrected for the bifocal metalens located at (0,30).

Figure 5(e) and (f) show the experimental results of transmittance and focusing efficiency of the X/Y bifocal metalenses located at (0,0) and (0,30), respectively. The MTFs are the Fourier transforms of the LSFs in the X-direction, as shown in Figure 5(g) and (h). That is, the gradient phase not only improves the transmission and focusing efficiency of X/Y bifocal metalens in large fields of view, but also corrects the aberration caused by oblique incident light.

**Figure 6:**
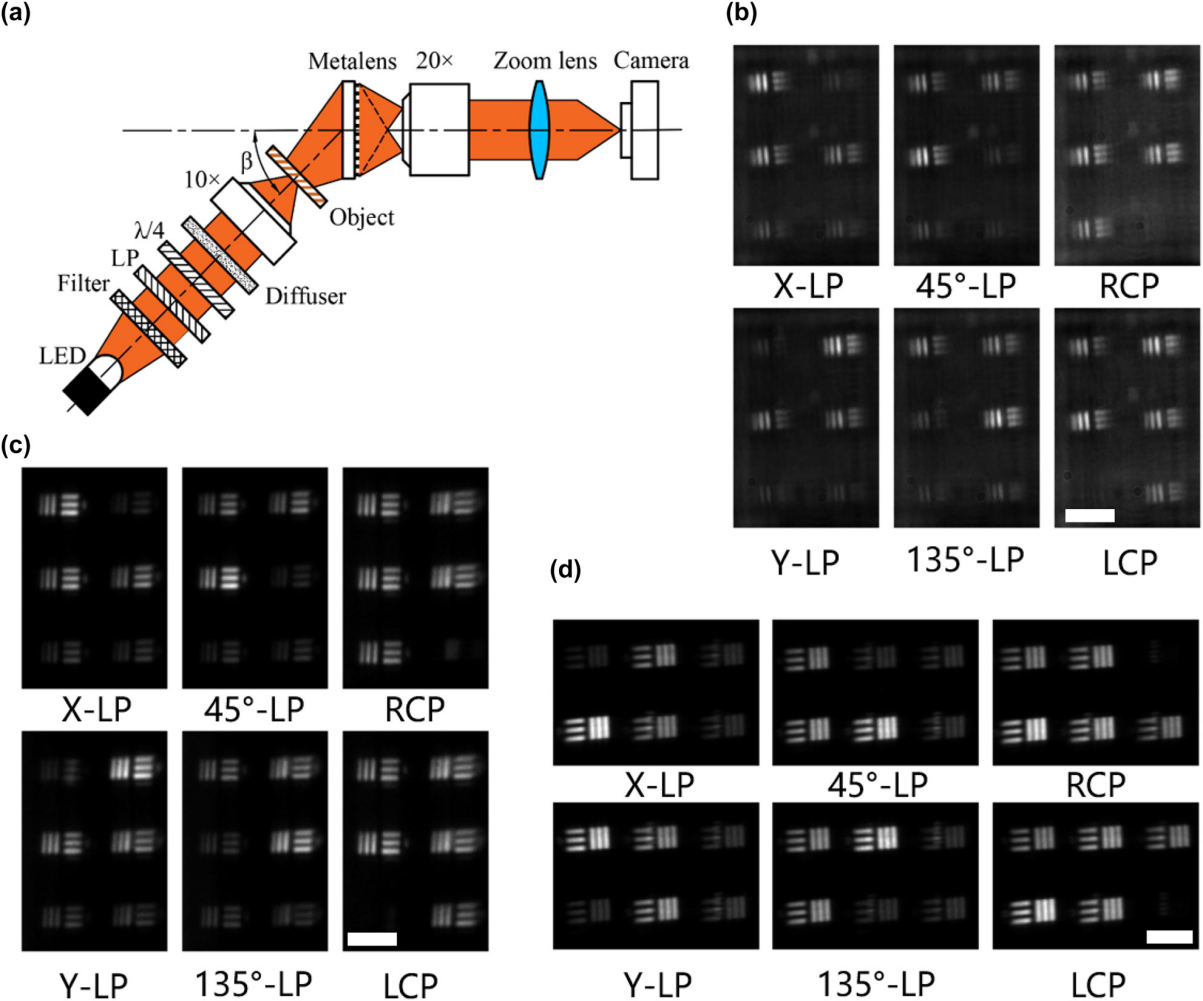
Experiment of polarization imaging by the BCEM. (a) Schematic diagram of the optical path used to test the imaging performance of the BCEM. (b)–(d) Full Stokes polarization imaging tests of sub eyes located at (0,0), (0,30), and (90,30), respectively. The results in (d) were tested with the BCEM rotated 90° counterclockwise.

**Figure 7:**
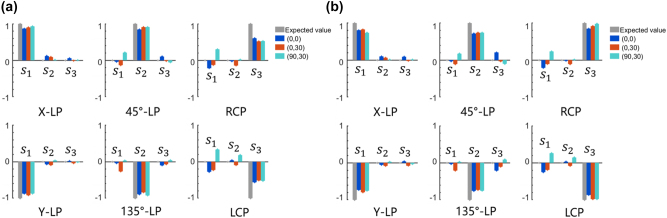
Characterization results of polarization imaging by the BCEM. (a) Stokes parameter reconstruction results of Figure 6(b)–(d). (b) Stokes parameter reconstruction results after normalizing the image intensity of Figure 6(b)–(d). Where gray, blue, red, and cyan bars represent the expected values, and experimental results of sub eyes located at (0,0), (0,30), and (90,30), respectively.

The imaging performance of the BCEM was tested using six stripes similar to the USAF resolution test chart, and the experimental optical path is shown in Figure 6(a). The light source uses LEDs with a wavelength of 780 nm, and the light is transformed into uniformly polarized light after passing through a filter (Hengyang Optics, China, HNIF-010-780-H-D25), a linear polarizer, a quarter-wave plate and a diffuser (C.F.Technology, China, DG10-1500). A 10× objective lens (Mitutoyo, M Plan Apo 10X/0.28) was used to focus light onto the resolution test chart, and then a 20× objective lens (Mitutoyo, M Plan Apo 20X/0.42), a zoom lens, and a camera were used to record the imaging of the BCEM. Figure 6(b) and (c) show the imaging tests of the sub eyes located at (0,0) and (0,30) with different polarized light, respectively. Due to the limitation of the two-dimensional experimental platform, the BCEM was rotated 90° counterclockwise before testing for the sub eye located at (90,30), and results are shown in Figure 6(d). It can be seen from Figure 6(b)–(d) that the intensity of the circularly polarized light image is smaller than that of the linearly polarized light image. In addition, the image of the polarization component orthogonal to the polarization state of the light source is also slightly visible. We think that the imperfection of the test results is due to the manufacturing error of the metasurface. Theoretically, the size of the elliptical pillar determines the phase shift and transmittance of the light, which further determines the independent control of the polarization component by the bifocal metalens. Therefore, the fabrication error of the metasurface is the main reason to affect its performance. To demonstrate the polarization information of the images obtained by BCEM in more detail, we extracted the Stokes parameters from the Figure 6(b) and (c), as shown in Figure 7(a) and (b) (method as in Supplementary Section 1). It is known from Figure 7(a) that the accuracy of the Stokes parameter reconstruction for linearly polarized light is higher than that for circularly polarized light by the three subeyes. For the reason that the intensity of the linearly polarized light image is larger than that of the circularly polarized light image. The reconstruction accuracy of circularly polarized light can be improved by normalizing the image intensity of the three bifocal metalenses in the sub eye (method as in Supplementary Section 1), as shown in Figure 7(b). Therefore, the full Stokes polarization imaging capability of the BCEM in a wide field of view was demonstrated experimentally.

## 5 Conclusions

In summary, we propose a BCEM that achieved full Stokes polarization imaging in a wide field of view. Similar to the natural compound eye, the BCEM consists of an array of sub eyes, and the orientation of the visual axis is different for each sub eye. Each sub eye consists of three bifocal metalenses which can achieve full Stokes polarization imaging for the target. Since the sub eyes located at different coordinates have different gradient phases, the BCEM can perform simultaneous full Stokes polarization imaging of targets in a wide field of view. The gradient phase improves the transmittance and focusing efficiency of the bifocal metalens at oblique incident light, as well as reduces the aberration caused by oblique incident light. Compared with the curved microlens array, the imaging plane of the BCEM can be completely coincident with the planar detector. In addition, the BCEM realizes the integrated design of lens and polarization device, which makes the bionic compound eye smaller in size and more integrated. The BCEM has great potential for endoscopy, industrial inspection, remote sensing, and underwater detection.

## Supplementary Material

Supplementary Material Details
