# Supplementary material for: Wide field of view and full Stokes polarization imaging using metasurfaces inspired by the stomatopod eye
Source: Nanophotonics. 2023 Feb 24;12(6):1137–46. doi: 10.1515/nanoph-2022-0712 (PMC11501549; doi:10.1515/nanoph-2022-0712)
Supplement: Supplementary file 1 — Supplementary Material Details [file j_nanoph-2022-0712_suppl.pdf]

Supplementary for:

Wide field of view and full Stokes polarization imaging using metasurfaces inspired by the stomatopod eye

Jianying Liu, Jinkui Chu*, Ran Zhang, Rui Liu, Jiaxin Fu
Key Laboratory for Micro/Nano Technology and System of Liaoning Province, Dalian University of Technology, Dalian, Liaoning, 116024, China.
*Corresponding authors. Email: chujk@dlut.edu.cn

Section 1.Stokes parameter reconstruction of the BCEM

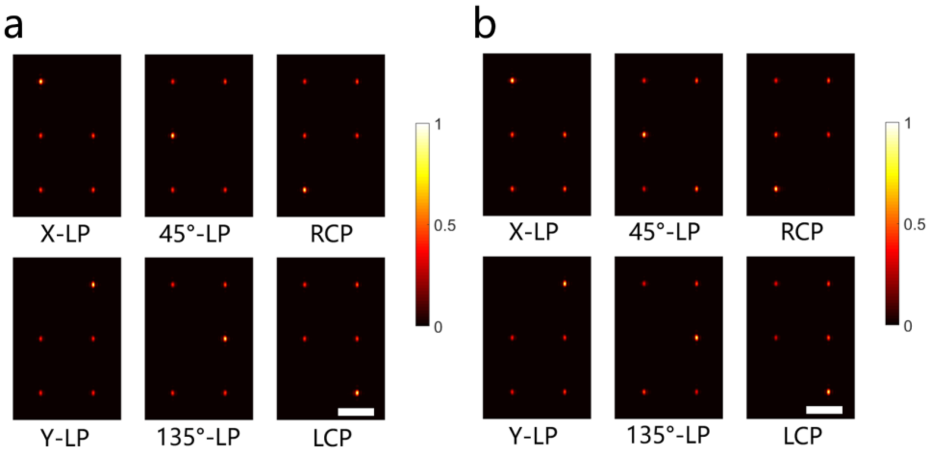

Fig. S1: Numerical simulation results. (a-b) The focal points of the subeyes located at (0,0) and (0,30) for incident light with different polarization states. Scale bar: 10 μm. The size of the subeye in the numerical simulation is 30 × 45 μm.

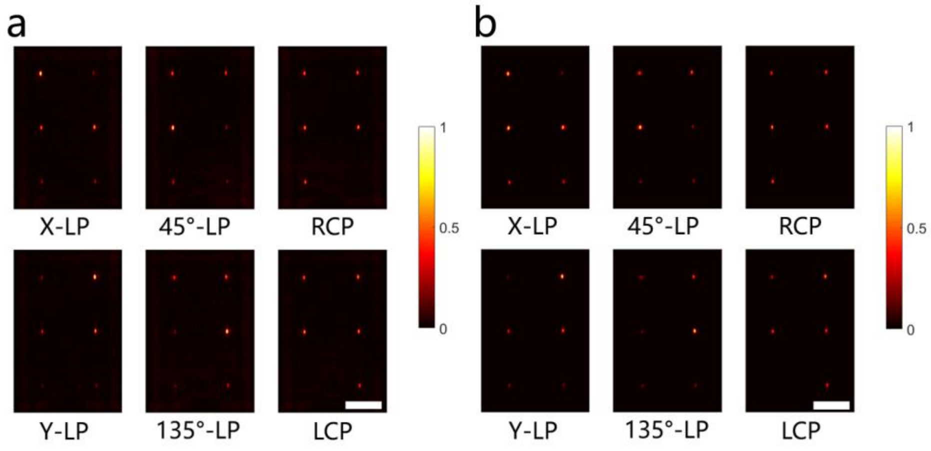

Fig. S2. Measurement results. (a-b) The focal points of the subeyes located at (0,0) and (0,30) for incident light with different polarization states. Scale bar: 40 μm. The size of the subeye is 120 × 180 μm. Due to the fabrication error of the metasurface, the focal spot intensity of the circularly polarized bifocal metalens is lower than that of the linearly polarized bifocal metalens.

The Stokes parameters based on the bifocal metalens can be expressed as:

$$S = \begin{bmatrix} S_0 \\ S_1 \\ S_2 \\ S_3 \end{bmatrix} = \begin{bmatrix} I \\ I_x - I_y \\ I_{45} - I_{135} \\ I_r - I_l \end{bmatrix} \quad (1)$$

Where $I = (I_x + I_y + I_{45} + I_{135} + I_r + I_l)/3$. I_x, I_y, I_{45}, I_{135}, I_r and I_l represent the light intensity of polarization components of X-LP, Y-LP, 45 °-LP, 135 °-LP, RCP and LCP, respectively. Equation (1) was used to reconstruct the Stokes parameters of the incident light and the results are shown in Figure S3.

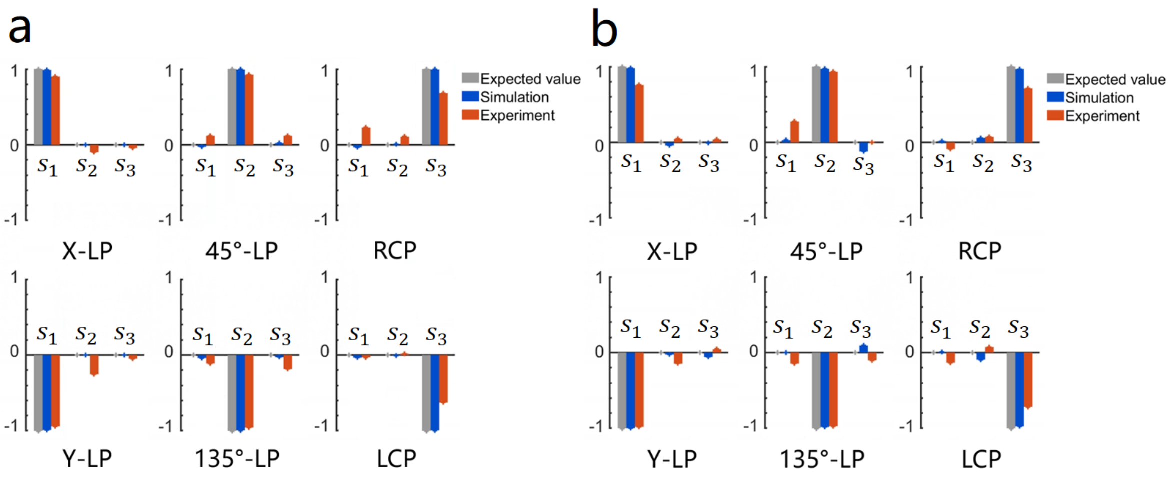

Fig. S3: (a-b) Stokes parameter reconstruction results for different input polarizations of the subeyes located at (0,0) and (0,30), respectively. Where the gray, blue and red bars represent the expected value, simulated and experiment results, respectively.

The errors in the Stokes parameters reconstructed from experiments are large due to the fabrication errors of the metasurface. The experimental results show that the transmittance of circularly polarized bifocal metalens is lower than that of linearly polarized bifocal metalens which results in smaller values of s_3 in the Stokes parameter.

In theory, the three bifocal metalenses in the subeye have the same transmittance. The intensities of the focused spot of the three bifocal metalenses are normalized using numerical processing as shown in Equations (2-3).

$$R_1 = (I_{45} + I_{135})/(I_x + I_y) \quad (2)$$

$$R_2 = (I_r + I_l)/(I_x + I_y) \quad (3)$$

The normalized light intensities $I'_x = I_x$, $I'_y = I_y$, $I'_{45} = R_1 I_{45}$, $I'_{135} = R_1 I_{135}$, $I'_r = R_2 I_r$, $I'_l = R_2 I_l$. The Stokes parameters are recalculated using Equation (1), as shown in Figure S4. The reconstruction accuracy of the Stokes parameter for circularly polarized light is improved after the light intensity normalization. But, the reconstruction accuracy for linearly polarized optical Stokes parameters is reduced. Therefore, the light intensity normalization method can compensate for the deficiencies of metal surface properties to some extent.

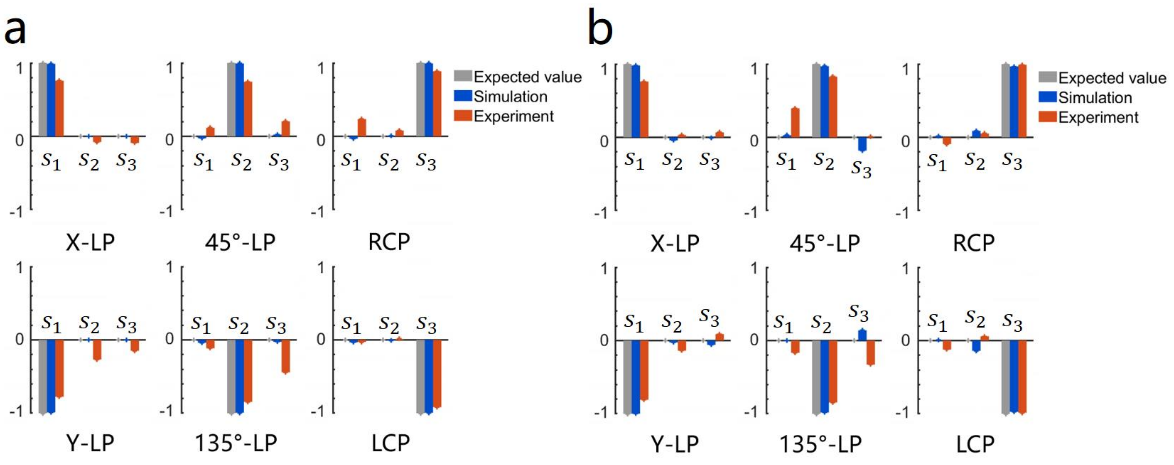

Fig. S4: (a-b) Stokes parameter reconstruction results for different input polarizations of the subeyes located at (0,0) and (0,30), respectively. Where the gray, blue and red bars represent the expected value, simulated and experiment results, respectively.

Section 2. Light shielding film fabrication

The fabrication process of the BCEM is the same as in the paper [1]. Here, we only describe the process of making Cr (Chromium) light shielding film after the metasurface is finished. First, a 2.5-μm-thick layer of UV photoresist (2035) is spin-coated on the metasurface, and the photoresist in the subeyes area is retained after the exposure and development process. Next, a 200 nm thick Cr is deposited on the metasurface by electron beam evaporation, where the photoresist on the subeyes is used as a protective film for the elliptical silicon pillars. Finally, the Cr is lifted off using acetone while the photoresist is removed from the subeyes. The fabrication of Cr light shielding film is completed.

Reference

[1] Q. Wei, B. Sain, Y. Wang, B. Reineke, X. Li, L. Huang, T. Zentgraf, "Simultaneous Spectral and Spatial Modulation for Color Printing and Holography Using All-Dielectric Metasurfaces," *Nano Letters*, vol. 19, no. 12, pp. 8964-8971, 2019.
